# Supplementary material for: Machine learning assisted vector atomic magnetometry
Source: Nat Commun. 2023 Sep 29;14:6105. doi: 10.1038/s41467-023-41676-x (PMC10541418; doi:10.1038/s41467-023-41676-x)
Supplement: Supplementary file 1 — Supplementary Information [file 41467_2023_41676_MOESM1_ESM.pdf]

Supplementary Information for Machine Learning Assisted Vector Atomic Magnetometry

X. Meng *et.al.**

Supplementary Note 1. Theoretical Model

In our simplified four-level model mentioned in Methods, we set the quantization axis to be the direction of the total magnetic field, which defines the $\boldsymbol{z'}$ axis of our theoretical coordinates (x', y', z'). Another set of coordinates (x, y, z) as shown in Fig.S1(a) is used which we call "optics" coordinates, since the laser propagates along $\boldsymbol{z}$ and its polarization angle α is relative to $\mathbf{x}$ in the (x, y) plane. The magnetic field direction is denoted by the altitude angle θ and the azimuth angle φ in the "optics" coordinates. Fig.S1(b) shows the relation between the two coordinates. In the theoretical coordinates, the polarization angle is β, relative to $\boldsymbol{x'}$ and with respect to a rotation about $\boldsymbol{k}(\boldsymbol{z''})$. We can derive the relation between the angles in the two coordinates as $\pi/2 - (\beta - \alpha) = \varphi$, with the assistance of a third set of coordinates (x'', y'', z'') for eye guiding.

The decomposition of the polarization components of light can be described as follows, for an ellipticity of ε:

$$\begin{pmatrix} E_{\hat{\sigma}+} \\ E_{\hat{\sigma}-} \\ E_{\hat{\pi}} \end{pmatrix} = \begin{pmatrix} \frac{(\cos\varepsilon\cos\theta+\sin\varepsilon)\cos\beta - i\sin\beta(\cos\varepsilon+\cos\theta\sin\varepsilon)}{\sqrt{2}} e^{-i\varphi} \\ \frac{(\cos\varepsilon\cos\theta-\sin\varepsilon)\cos\beta + i\sin\beta(\cos\varepsilon-\cos\theta\sin\varepsilon)}{\sqrt{2}} e^{i\varphi} \\ (-\cos\beta\cos\varepsilon + i\sin\beta\sin\varepsilon)\sin\theta \end{pmatrix} \tag{1}$$

Then we can write down the effective Hamiltonian after rotating wave approximation (RWA):

$$H_{eff} = \begin{pmatrix} \Omega_L & 0 & 0 & \Omega_R E^*_{\hat{\sigma}-} \\ 0 & 0 & 0 & -\Omega_R E^*_{\hat{\pi}} \\ 0 & 0 & -\Omega_L & \Omega_R E^*_{\hat{\sigma}+} \\ \Omega_R E_{\hat{\sigma}-} & -\Omega_R E_{\hat{\pi}} & \Omega_R E_{\hat{\sigma}+} & -\Delta - \Delta_m \cos\omega_m t \end{pmatrix} \tag{2}$$

where Δ is the one-photon detuning and Ω_R, Ω_L represent the Rabi frequency of the laser and the Larmor frequency (proportional to the amplitude of the total magnetic field) of the atoms respectively. ω_m is the modulation frequency of the laser and Δ_m is the modulation amplitude. The atomic coherences can be found by solving the density matrix equation:

$$\frac{\partial\rho}{\partial t} = -\frac{i}{\hbar}[\hat{H}, \rho] + (\Gamma_{\text{rel}} + \Gamma_{\text{rep}})\,\rho \tag{3}$$

where Γ_{rel} describes the decoherences including the spontaneous decay and dephasing etc., and Γ_{rep} describes the repopulation of the ground state [1]. Due to the periodicity of the system

*yxiao@fudan.edu.cn

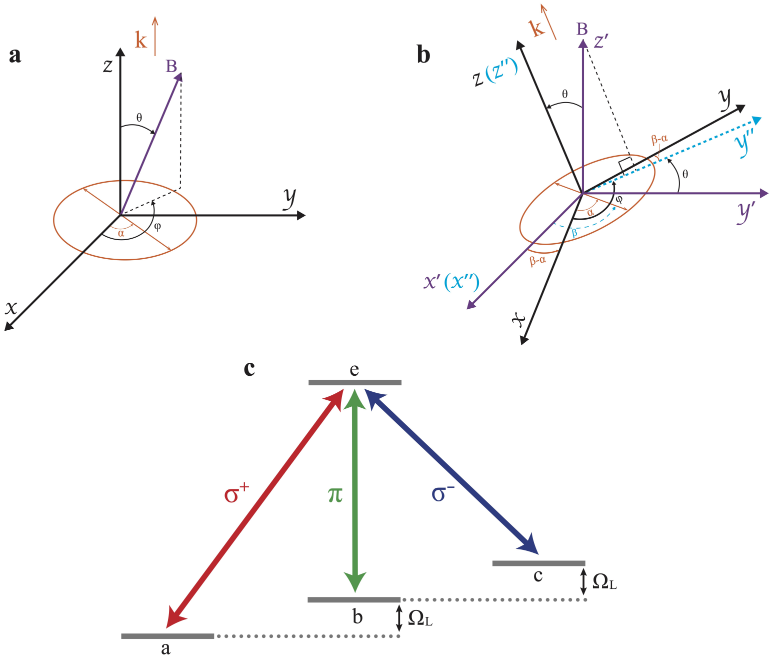

Fig.S 1. ***Coordinates in theory and simplified four-level model.*** *(a) The "optics" coordinates (x, y, z) where the* ***z*** *axis is the direction of the light propagation. The magnetic field direction is denoted by the altitude angle θ and azimuthal angle φ. In the light polarization plane, α denotes the direction of the principle axis of the elliptically polarized light. (b) The theory coordinates (x', y', z') where* ***z′*** *is the quantization axis defined by the total magnetic field's direction. β is the polarization angle in this coordinate system. There is a clear relation $\pi/2 - \beta + \alpha = \varphi$ between the two coordinates. A third coordinate system (x'', y'', z'') is used for eye guiding. (c) The simplified four-level atomic energy level.*

under frequency modulation, the coefficients of a Fourier expansion of the density matrix can be identified using the Floquet technique where $\rho(t)$ is expanded in harmonics of the modulation frequency ω_m:

$$\rho(t) = \sum_{n=-\infty}^{\infty} \rho^{(n)} e^{in\omega_m t} \tag{4}$$

We measure the polarization rotation signal of the light after it passes through the atomic sample. The rotation signal can be derived directly from the atomic coherences for an optically thin medium:

$$\begin{aligned} Rotation \propto & \Big(\cos\beta \cos\varphi(\cos\theta \sin\varepsilon - \cos\varepsilon) + \sin\beta \sin\varphi(\cos\varepsilon \cos\theta - \sin\varepsilon) \Big) Re[\rho_{ae}] \\ & + \Big(\sin\beta \cos\varphi(\cos\theta \cos\varepsilon + \sin\varepsilon) + \cos\beta \sin\varphi(\cos\theta \sin\varepsilon + \cos\varepsilon) \Big) Im[\rho_{ae}] \\ & + \Big(-\cos\beta \cos\varphi(\cos\theta \sin\varepsilon + \cos\varepsilon) + \sin\beta \sin\varphi(\cos\theta \cos\varepsilon + \sin\varepsilon) \Big) Re[\rho_{ce}] \\ & + \Big(\cos\beta \sin\varphi((\cos\theta \sin\varepsilon + \cos\varepsilon)) + \sin\beta \cos\varphi(\cos\theta \cos\varepsilon + \sin\varepsilon) \Big) Im[\rho_{ce}] \\ & + \sqrt{2} \Big(\sin\beta \sin\theta \cos\varepsilon \cdot Im[\rho_{be}] - \cos\beta \sin\theta \sin\varepsilon \cdot Re[\rho_{be}] \Big) \end{aligned} \tag{5}$$

Then the different harmonics, i.e., the demodulated signal in the optical rotation can be obtained, since the atomic coherences contain the oscillations at ω_m and its harmonic frequencies. It can be seen that, the full vectorial information of the magnetic field has been encoded in the optical rotation signals.

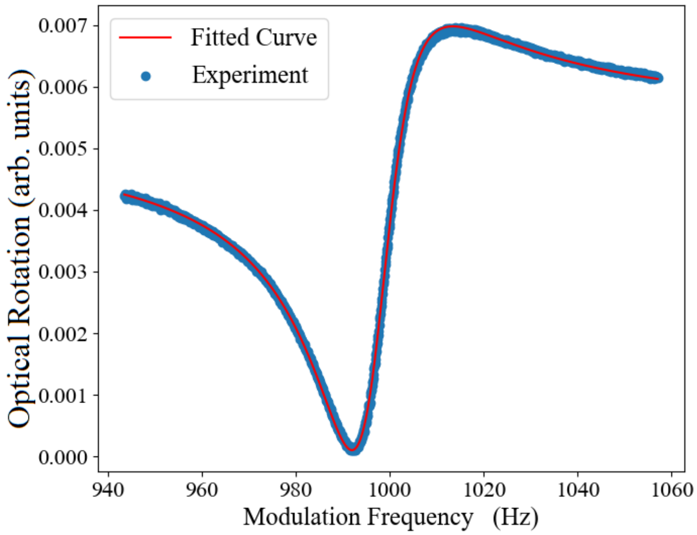

Fig.S 2. ***Exemplary NMOR spectrum for magnetic field calibration.*** *The magnitude of a vector synthesized magnetic field is found to be 143.98 nT corresponding to a Larmor frequency of 1007 Hz, through curve fitting using a linear superposition of a Lorentz absorption and dispersion function. The laser modulation frequency was slowly swept.*

Supplementary Note 2. Calibration of Magnetic Field

In the Methods section, we describe the magnetic field calibration procedure following reference [2]. Here, we provide more details. Fig.S4 shows the distribution of the measured magnetic field values around the set magnetic field magnitude before and after the calibration, indicating improved accuracy.

We note that, the (X, Y, Z) coordinates used in the field calibration are the one we use in denoting the experimentally measured magnetic field, but is in general slightly deviated from the "optics" coordinates (x, y, z) in the previous section. In particular, the $\boldsymbol{z}$ axis in the "optics" coordinates is along the wave vector of the laser field, and the $\boldsymbol{Z}$ axis we use to define the measured θ value of $\mathbf{B}$ is along the magnetic field produced by the set of Helmholtz coil oriented approximately (but not exactly due to experiment imperfections) along $\mathbf{k}$. Also, the $\boldsymbol{x}$ axis in the "optics" coordinates and the $\boldsymbol{X}$ axis in the calibration experiment are not the same, with the latter close to the axis of the coil aligned approximately along that direction. We emphasize that, such a difference does not affect the experimental magnetometer results given by the machine learning (ML) approach, since the master equation is not used at all in the ML model. This further showcases that ML-based methods do not rely on any explicit physics models.

Supplementary Note 3. Optimization of single-axis magnetometer sensitivity

Before measuring a vector magnetic field, the performance of the single-axis magnetometer is optimized in three orthogonal directions respectively. We set the laser modulation range to be 400 MHz and the center frequency of the laser is 200 MHz red-detuned from the ^{87}Rb D_1 $|F = 2 > \to |F' = 1 >$ transition. In most NMOR experiment, the input laser is linearly polarized and the zero magnetic field resonance is used, where, for a frequency modulated laser beam, the resonance amplitude at $\Omega_L = \omega_m$ may be small. Since we need to use this side resonance for vector magnetometry, an elliptical polarized laser beam has been adopted to increase this side resonance's amplitude, and furthermore its elipticity was optimized for comparable sensitivities in three orthogonal directions. Considering the symmetry, we set the long axis of the elliptical polarization to be along the angular bisector of **X** and **Y**. The ellipticity ϵ was tuned to be about 0.11π. Fig.S5 shows the magnetic resonance measured for each set of coil separately when sweeping the current in the coil. It can be seen that at $\Omega_L = \omega_m$ (the resonance near 140 nT in the figure), the demodulated optical rotation signals at both ω_m and $2\omega_m$ exhibit sizable slopes in all three directions.

Supplementary Note 4. Sensitivity given by Neural Network

In our scheme, a trained NN uses the in-phase and quadrature of the optical rotation signal at the first and second harmonics of ω_m as its input and gives the full vector information of the magnetic field at the output. After training, all weights in the NN layers are fixed and there is a determined relationship between the input signals and the output magnetic field parameters, which can be illustrated by the functions $O_j(M_i|i = 1, 2, 3, 4)$. Here $O_j(j = 1, 2, 3)$ represents one of the polar component of the magnetic field and M_i is the demodulated signal (or meter). Fluctuations in the input signal are then translated into fluctuations of the polar components of the magnetic field:

$$\delta O_j = \sum_i \frac{\partial O_j}{\partial x_i} \delta x_i \tag{6}$$

In the experiment we conduct measurements of the optical rotation signals for one minute, and the four signals X_1, Y_1, X_2, Y_2 at each time point are sent to a trained NN which then outputs a (B, θ, φ) set for that time point. Consequently, we obtain an effective time trace for B, θ and φ respectively. Then, fast-Fourier-transforming (FFT) is done for the three time traces and the noise spectrum is produced. To obtain the sensitivities, the frequency response of the atoms must be taken into account. We measure the response curve at a fixed **B** near 140 nT by adding a small AC magnetic field to introduce small oscillations to B, θ and φ. We then record the four oscillatory time trace of the optical rotation signals, and transform them to oscillatory time trace of B, θ and φ via the trained NN, in the same way as we obtain the noise spectrum of the field components above. The amplitude of the oscillation at the frequency of the applied AC field is then extracted in each polar components of **B**. By repeating the measurements at several frequencies of the small AC magnetic field, we get the scattered data points around the (fitted) response curve as shown as Fig.S6. Here the solid line curve is a fit with the function $R(f) = 1/\sqrt{1 + f^2/BW^2}$, where the bandwidth BW is about 14 Hz.

Supplementary Note 5. Sensitivity of vector magnetometer at different directions

In the main text of the paper, we show the sensitivity of our vector magnetometer at a particular angle $\theta = 63.435^\circ, \varphi = 60^\circ$ as an example. Here, we give the sensitivities on the three polar field components in all directions, for a change of the field vector at 11 Hz imposed on a field about 140 nT pointing along the indicated direction, as shown in Fig.S7. The sensitivities are derived from the NN-aided noise analysis method as in Fig.4a of the main text. It can be seen that the sensitivity levels have small variations in most parameter space, except that near $\theta = 0$ and 180° the sensitivity for φ is worse, as explained in the main text.

Supplementary Note 6. Proposed angled multi-pass scheme to lift degeneracy and remove dead zone

One problem of the current experiment scheme is that the signals are degenerate for φ and $\varphi + \pi$. Another issue is the dead zone for φ when $\mathbf{B}$ is nearly aligned with $\mathbf{k}$ of the laser. Here we propose to address these problems using an angled three-pass scheme as drawn in Fig.S8, where the laser is reflected back to the atomic sample twice at an angle. Our numerical simulation shows that, in comparison to the single-pass scheme, the multi-pass scheme can indeed lift the degeneracy and remove the dead zone for φ (see Fig.S9 and Fig.S10 respectively), while remaining to be a single-beam configuration. We note that as long as $\mathbf{k}$ has both horizontal and vertical components, these two problems about φ can be solved.

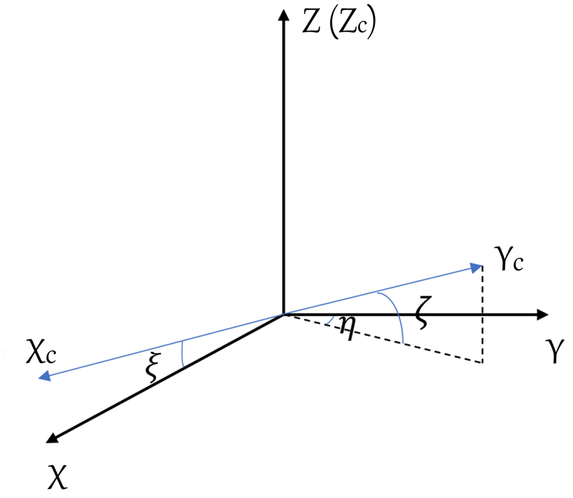

Fig.S 3. ***Illustration of the non-orthogonal experiment coils.*** *$\mathbf{X_c}, \mathbf{Y_c}, \mathbf{Z_c}$ represent the directions of the magnetic field generated by the three sets of Helmholtz coils. $\mathbf{X_c}$ is in the $\mathbf{X}$-$\mathbf{Z}$ plane and the deviations from an ideal coordinate system (X, Y, Z) are described by the three angles ξ, η, ζ.*

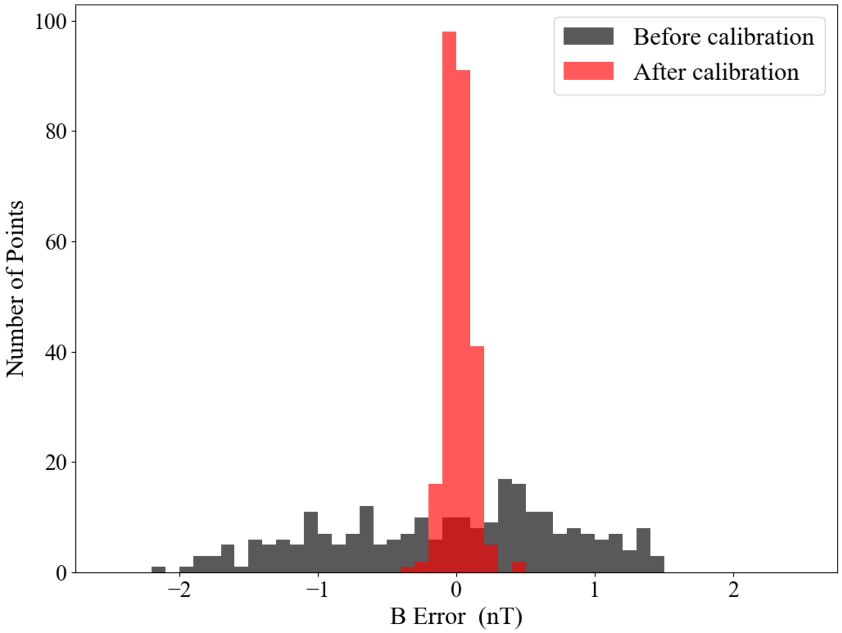

Fig.S 4. ***Distribution of measured magnetic field values around the set magnetic field magnitude.*** *Magnetic field error before and after the calibration. Before calibration the magnetic field deviates considerably from the intended set value, and consequently, the field direction has a relatively wide distribution. Through the calibration process we found the non-orthogonal angles and residual magnetic field* $(\xi, \eta, \zeta, B_{X_0}, B_{Y_0}, B_{Z_0})$ *to be* $(0.084°, 2.06°, 1.68°, 17.78pT, -538.44pT, 48.78pT)$. *With these parameters we can set the magnetic field with enhanced accuracy.*

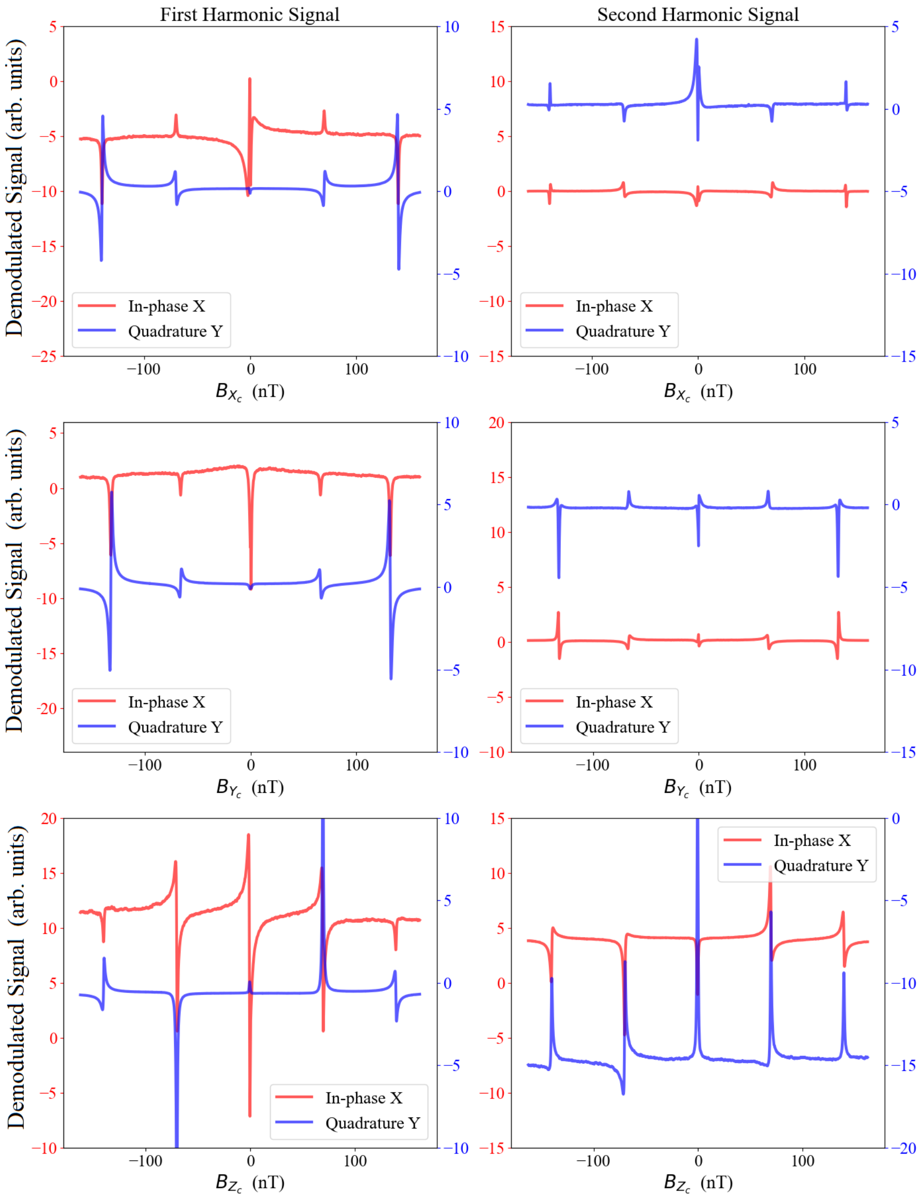

Fig.S 5. ***NMOR signal with elliptically polarized light in three orthogonal directions.*** *The magnetic resonance we use for our vector magnetometer is at $\Omega_L = \omega_m$. By adjusting the ellipticity of the laser, we can obtain similar slopes at the center of this resonance in the three directions. In the figures, the red (blue) curves correspond to the red (blue)* $\boldsymbol{y}$*-axis.*

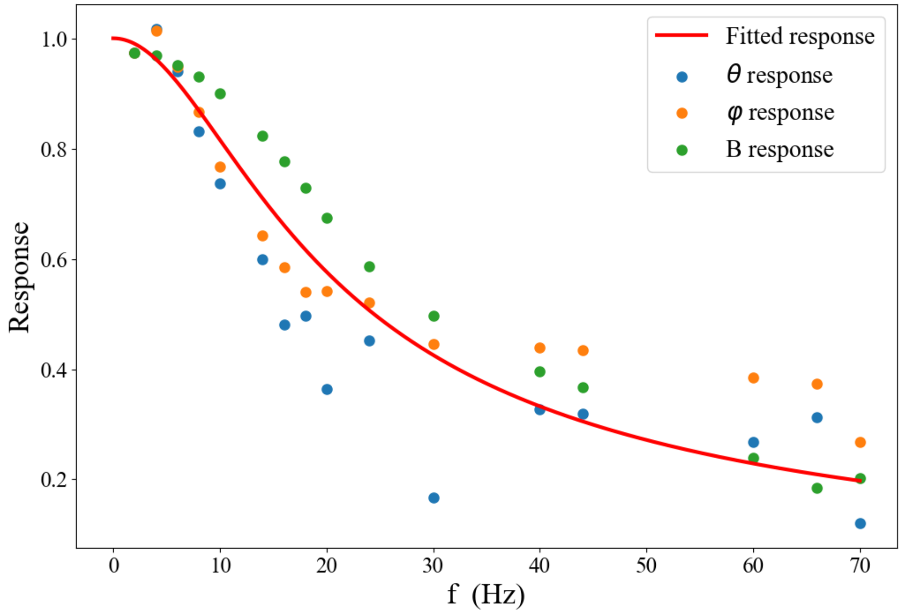

Fig.S 6. ***Measured frequency response of the vector magnetometer with the aid of NN.*** *The magnetic field is at the direction of* $\theta = 63.435^\circ$, $\varphi = 60^\circ$. *The applied small AC field is along* $\mathbf{X_c}$ *and* $\mathbf{Z_c}$ *direction. The fitted bandwidth is about 14 Hz.*

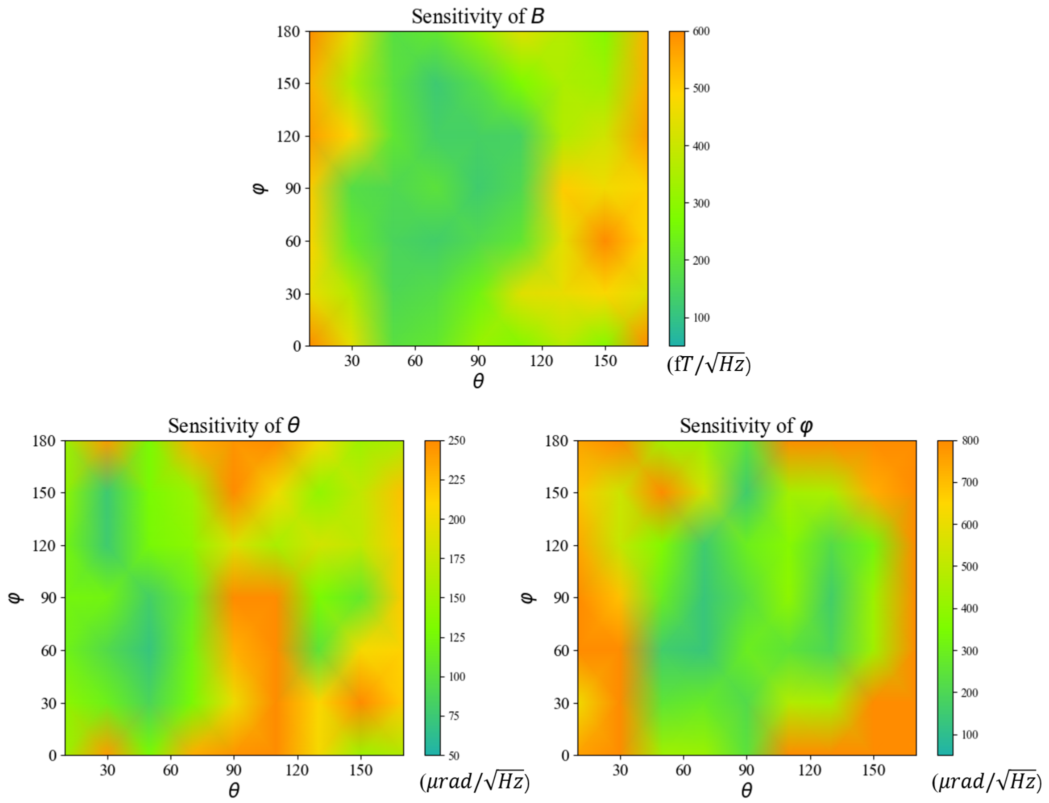

Fig.S 7. ***Sensitivity of the NN aided vector magnetometer along different field directions.*** *The sensitivity of the field amplitude B ranges from 100 to 600* $fT/\sqrt{Hz}$ *and the sensitivity of* θ *is about* $100 \sim 200$ $\mu rad/\sqrt{Hz}$. *Excluding the regions where* θ *is near 0 and* 180°, *the sensitivity of* φ *is about 200* $\mu rad/\sqrt{Hz}$.

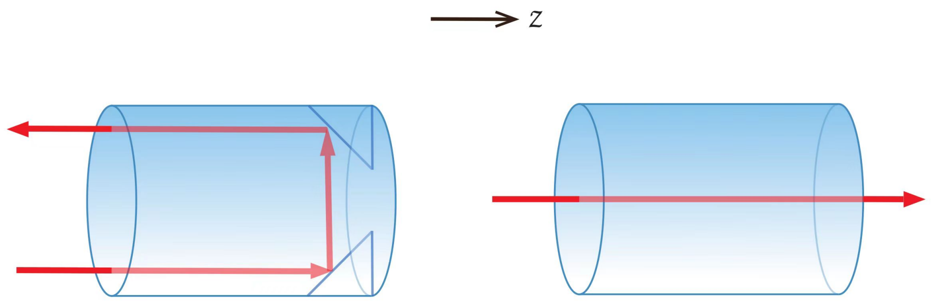

Fig.S 8. ***Proposed three-pass scheme and the current single-pass scheme.*** *We propose to use the three-pass scheme in the future to lift the degeneracy between the field directions* φ *and* $\varphi + \pi$, *as well as to remove the dead zone for* φ *when* $\boldsymbol{B}$ *is nearly aligned with* $\boldsymbol{k}$. *The red arrow is the laser beam, and the cylinder is the atomic vapor cell.*

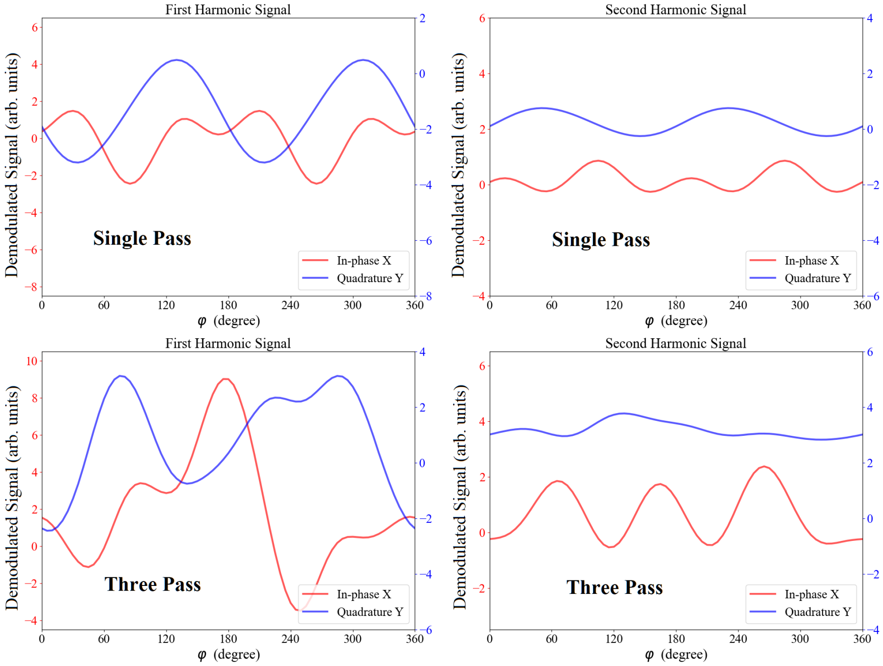

Fig.S 9. ***Calculated NMOR signals for the single-pass and three-pass schemes.*** *The three-pass scheme can break the degeneracy for the field directions between φ and $\varphi + \pi$. In the simulation, $\theta = 60°$. In the figures, the red (blue) curves correspond to the red (blue) $\boldsymbol{y}$-axis.*

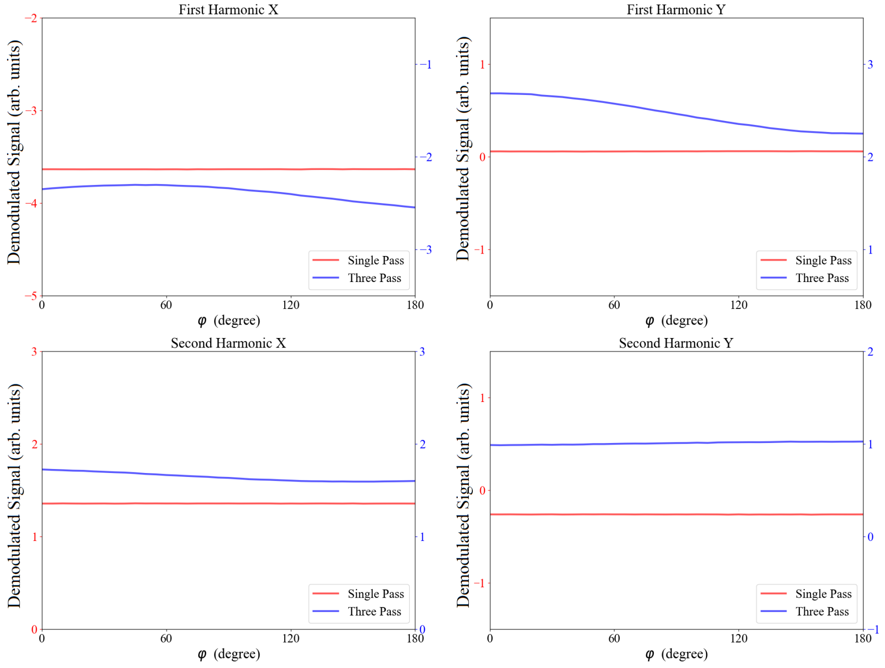

Fig.S 10. ***Calculated NMOR signals for the single-pass and three-pass schemes.*** *The three-pass scheme can remove the dead zone for φ when* $\mathbf{B}$ *is nearly aligned with* $\mathbf{k}$*. In the simulation, $\theta = 1°$. In the figures, the red (blue) curves correspond to the red (blue)* $\mathbf{y}$*-axis.*

Supplementary References

[1] Jin, S., Bao, H., Duan, J., Lu, X., Wang, M., Zhao, K.-F., Shen, H., & Xiao, Y. Adiabaticity in state preparation for spin squeezing of large atom ensembles. *Photonics Res.* **9**, 2318 (2021).

[2] Thiele, T., Lin, Y., Brown, M. O., & Regal, C. A. Self-calibrating vector atomic magnetometry through microwave polarization reconstruction. *Phys. Rev. Lett.*, **121**, 153202 (2018).
